# Automatic joint inflammation estimation based on regression neural networks

**DOI:** 10.1002/mp.70010

**Published:** 2025-09-22

**Authors:** Yanli Li, Dennis A. Ton, Denis P. Shamonin, Monique Reijnierse, Annette H. M. van der Helm‐van Mil, Berend C. Stoel

**Affiliations:** ^1^ Division of Image Processing Department of Radiology Leiden University Medical Center Leiden The Netherlands; ^2^ Department of Rheumatology Leiden University Medical Center Leiden The Netherlands; ^3^ Department of Radiology Leiden University Medical Center Leiden The Netherlands

**Keywords:** deep learning, inflammation assessment, metacarpophalangeal, metatarsophalangeal, MRI, rheumatoid arthritis, wrist

## Abstract

**Background:**

Quantitative assessment of inflammation from hand and forefoot MRI scans is crucial for evaluating the severity, progression, and treatment response in inflammatory disease like rheumatoid arthritis (RA). Traditionally, this relies on visual evaluation of signs like bone marrow edema (BME), tenosynovitis, and synovitis, which is time‐consuming, subjective, and prone to inherent inter/intra‐reader variability.

**Purpose:**

This study aims at an automatic DL‐based MRI analysis of inflammatory signs in RA system for inflammation assessment to facilitate related diagnoses and studies.

**Methods:**

We developed an **A**utomatic **D**L‐based **M**RI analysis of **I**nflammatory signs in **RA** (ADMIRA) system for inflammation assessment, using pre‐ and post‐processing alongside DL models to estimate inflammation scores from fat saturated, contrast‐enhanced T1‐weighted MRI scans of 2254 subjects across four study populations. These MRI scans include three different anatomical sites, wrist, metacarpophalangeal (MCP) and metatarsophalangeal (MTP) joints, as the objects for inflammation assessment. The scans were divided into training, monitoring, testing and validation sets to ensure robust performance evaluation, using Pearson's correlation coefficients and Intra‐class correlation coefficients. A revised class activation mapping (CAM) algorithm was used to validate the DL model's reliability, illustrating its inference process.

**Results:**

The system achieved mean R/ICCs of nearly 0.9 for synovitis and tenosynovitis on test sets and 0.8 on the validation set, with slightly lower scores for BME (0.8 and 0.7, respectively). This system presents a performance close to human experts on the same datasets. Meanwhile, the visualization results indicate the DL models have a inference process consistent with expert knowledge.

**Conclusions:**

Results show that ADMIRA provides accurate, expert‐level inflammation estimation, particularly for synovitis and tenosynovitis, offering a fast, reliable alternative to manual methods for RA monitoring and analysis. We expect that this automatic method could help to reduce labor costs and improve the efficiency of diagnosis in the future.

## 1 INTRODUCTION

Detecting inflammation in the joints of hands and forefeet is essential for recognizing patients at risk of developing inflammatory diseases and for improving patient outcomes. For instance, it helps diagnose rheumatoid arthritis (RA) at an early stage, especially in combination with serological markers.^1^, ^2^, ^3^ Early diagnoses allow timely treatment, which can improve long‐term patient outcomes with higher chances of sustained remission without drugs and improved quality of life.^4^, ^5^

Currently, MRI is the most sensitive imaging method for detecting joint inflammation.^6^ In a research setting, MRI scans are assessed semi‐quantitatively by visual scoring systems such as the Rheumatoid Arthritis Magnetic Resonance Imaging (MRI) Scoring (RAMRIS) system.^7^ Using such scoring systems, trained readers examine multiple tendons or tendon groups, bones and synovia, quantifying the thickness of peritendinous effusion or synovial proliferation and the volume of bone marrow edema. Through this process, such scoring systems provides (semi‐)quantitative measurements of multiple image biomarkers, including those describing inflammation severity. However, this is a laborious and difficult task that requires significant time investment, is prone to inter‐reader variability and demands rigorous training to ensure accuracy.

To obtain fast and accurate inflammation measurements, an automatic inflammation estimation method could help alleviate labor and time costs, avoiding inter‐/intra‐reader disagreement, and ultimately improve the diagnosis, monitoring and prediction of inflammatory diseases. However, the automatic quantification of the inflammation in MRI scans is challenging and under‐investigated. An accurate, fast and automatic inflammation estimation system that simulates the principles of visual scoring requires not only precise annotations or segmentation of regions of interest (ROIs) on all related anatomical structures in the hands and feet, but also algorithms to identify relevant high‐intensity regions indicating of inflammation. In previous studies, Chand et al.^8^ proposed a segmentation‐quantification method to quantify synovitis in MRI scans of 38 patients, following a process of segmenting ROIs and then quantifying the high‐intensity pixels in these ROIs. Similarly, Aizenberg et al.^9^, ^10^ developed an automatic framework to quantify tenosynovitis and bone marrow edema (BME) in wrist MRI scans of 563 patients, based on atlas‐based region annotations and intensity measurements in those anatomical structures. Subsequently, with advances in deep learning (DL), Shamonin et al.^11^ developed a different framework using DL‐based segmentation to obtain annotations for tendons, bones and other anatomical structures. Combined with a fixed threshold, the method measures the volume of high‐intensity pixels in the ROIs, in order to quantify (teno‐)synovitis and BME on wrist MRI scans of 1225 patients. Yiu et al.^12^ developed a similar segmentation‐quantification framework on MRI scans of 80 RA patients, segmenting anatomical structures with DL models and quantifying high‐intensity pixels. To mitigate the dependency on accurate annotations of anatomical structures, Mao et al.^13^ proposed a method for estimating synovitis in MRI scans of 47 RA patients using ROIs instead of annotations. In this study, a classification DL model with different classes that represents different inflammation severity was trained on a small group of manually annotated regions of interest (ROIs) in MRI scans to quantify synovitis and conduct the inference process using ROIs, generated by an unsupervised image segmentation preprocessing rather than accurate annotations or the manual selected ROIs in.^14^

Although these methods offer intuitive explainability and user‐interaction, they require accurate annotations or segmentation of ROI, which are time‐consuming processes and can lead to propagation of errors. Furthermore, when extending the system for analyzing MRI scans of other anatomical region (such as scans at the level of the metacarpophalangeal joints in the hand or metatarsophalangeal joints in the feet), it takes considerably more time to obtain accurate ground truth annotations than ground truth visual scores.

A solution to this limitation is the development of an end‐to‐end automatic framework that directly estimates inflammation scores using DL models without requiring annotations. A framework of mimicking the visual scoring process can be trained on a small group of MRI scans and then applied to other MRI scans without the need for ground truth annotations.

This type of DL‐based automatic framework, which quantifies structural pathological symptoms or image biomarkers, have already been widely investigated in general medical image analysis. For semi‐quantification tasks that aim at discrete grades or categories, the methods were usually designed to apply classification instead of regression, such as Gleason scoring of prostate cancer in histopathology images,^15^, ^16^, ^17^ grading of ulcerative colitis in endoscopic images,^18^ diabetic retinopathy grading in eye fundus images^19^ and knee osteoarthritis severity classification in MRI scans.^20^ For quantification tasks that output continuous scores, regression neural networks are preferred, such as ventricle function indices estimation^21^ and arthritis activity scoring based on ultrasound,^22^ bone erosion scoring based on x‐rays,^23^ Agatston scoring,^24^ coronary calcium scoring^25^, ^26^ and systemic sclerosis scores^27^ based on CT scans. In terms of applications on MRI scans, studies such as grading of abnormalities^28^ and osteoarthritis severity grading in knee MRI^29^ provided references in building DL‐based frameworks for scoring image biomarkers.

Since these studies were not designed for estimating joint inflammation, it is difficult to transfer these ideas or models to our domain. For instance, their model architecture may require a specific input shape or image features, which are not feasible in our study. The closest study to our work is a DL‐based classification for erosion, synovitis and osteitis in hand MRI scans,^30^ which follows a two‐step process including semi‐automatic localization for regions of interests (ROIs) and DL‐based estimation based on ROIs. The semi‐automatic localization for ROIs requires human experts to put 3D landmarks corresponds to the 3D centers of anatomical ROIs, slowing down the process and introducing extra labor and time investment. Meanwhile, the DL‐model based on ROIs was pretrained through a 3D video classification task and limited to 3D MRI scans with high resolution on all axes, which are not always available. DL models for 3D MRI scans requires considerable amount of data for thorough training and more hardware memory for development and implementation, and will not be useful and generalizable if the cost is even higher than manual visual scoring.

Furthermore, some tasks require more than one MRI scan as input, that is, multi‐view or multi‐site inputs (e.g., in systemic diseases with symptoms in different anatomical regions as RA). For these tasks, an ideal method should be capable of coping with memory constraints and fusing the information from multiple inputs. In the specific case of our study, it is important that information from coronal and transversal views are fused, in much the same way human experts do, while performing visual scoring. Both coronal and transversal views are 3D images, but with the highest resolution in either the coronal or transversal plane, respectively.

To overcome these challenges, we propose a DL‐based framework that includes automatic preprocessing to select the most informative slices and a DL model to extract and integrate information from both views. This automatic framework utilizes the intensity difference between inflamed and normal tissues to enhance the information density, reduce noise and fuse the information from coronal and transversal scans using cross‐attention blocks in the DL architecture. Through learning from the visual scoring system RAMRIS that assess the joint inflammation for RA, this automatic DL‐based MRI analysis of inflammatory signs in RA (ADMIRA) system is expected to assess joint inflammation based on hand and forefeet MRIs.

The layout of this paper is as follows. First, we introduce our MRI materials and define the task. Subsequently, we clarify the pre‐processing and the architecture of the DL model. We then present experiments evaluating the proposed method for scoring synovitis, tenosynovitis and BME in MRI scans of the wrists, metacarpophalangeal (MCP) and metatarsophalangeal (MTP) joints, collected from 2279 subjects. Finally, we discuss and summarize the limitations and advantages of the proposed methods in the concluding chapters.

## 2 METHODS

### 2.1 Data

To develop and assess the ADMIRA system, we utilized a database with four populations with different severity of joint inflammation and containing MRI scans of three anatomical regions: wrists, metacarpophalangeal (MCP), and metatarsophalangeal (MTP) joints from a period of more than ten years. In total, 2279 subjects (patient characteristics in Table 1) were collected for training the DL models and validating the whole method. Informed consent was given by all patients, referring to the LUMC protocol reference numbers: B19.008 and P11.210. The database consisted of the following four populations: 1247 subjects with early onset arthritis (EAC), 620 patients with clinically suspected arthralgia (CSA), 177 healthy controls and 236 CSA patients with longitudinal MRI scans from the TREAT EARLIER (TE) trial (not included in the CSA group).

**TABLE 1:** Characteristics of subjects.

Characteristic	EAC	CSA	Healthy controls	TREAT EARLIER trial
No. of patients	1226	616	177	236
Mean age (y) (first time point for longitudinal data) ± SD	56.5 ± 15.6	43.2 ± 12.6	49.8 ± 15.8	46.7 ± 11.9
Female (%)	698 (56.9)	473 (76.8)	136 (70.5)	154 (65.3)
Total RAMRIS inflammation score, median [IQR]	11 [4.5 – 21.5]	2 [1 – 5]	2 [0.5 – 4.5]	4.5 [3 – 8]
Synovitis score, median [IQR]	4.5 [1.5 – 8]	1 [0 – 2]	0.5 [0 – 2.5]	2 [1 – 4]
Tenosynovitis score, median [IQR]	2.5 [0 – 6]	0 [0 – 1]	0 [0 – 0]	1 [0 – 2.5]
Osteitis score, median [IQR]	3 [1 – 6.5]	0.5 [0 – 1.5]	1 [0 – 2]	1 [0 – 2]

As presented in Table 2, subjects were scanned with an ONI MSK Extreme 1.5T extremity MRI scanner (GE Healthcare, Waukesha, WI, USA) with a 100 mm coil. After intravenous injection of 0.1 mmol/kg Gd‐chelate (gadoteric acid, Guerbet, Paris, France), a T1‐weighted fast spin‐echo sequence with frequency‐selective fat saturation was obtained in the axial plane with a repetition time of 570 ms, echo time of 7 ms, acquisition matrix 320×192, echo train length 2, slice thickness of 3 mm, and a slice gap of 0.3 mm. Axial images were reconstructed with an image size of 512×512×20±5 voxels (voxel size: 0.195×0.195×3.0 mm). Coronal images were reconstructed with an image size of 512×512×20±5 voxels (voxel size: 0.195mm×0.195mm×2.2 mm).

**TABLE 2:** Technical parameters of the hand and forefoot MRI scans.

MRI Parameter	Transversal (axial) scan	Coronal scan
In‐plane matrix	320x192	364x224
Repetition time (ms)	570	650
Echo time (ms)	7	17
Echo train length	2	2
Slice thickness (mm)	3	2
Slice gap (mm)	0.3	0.2
Fat saturation	Frequency‐selective fat saturation applied	
Scanner	ONI MSK Extreme 1.5T extremity MR scanner (GE Healthcare, Waukesha, WI, USA)	
Coil	100 mm coil	
Contrast	intravenous injection of 0.1 mmol/kg Gd‐chelate (gadoteric acid, Guerbet, Paris, France)	
Other	No acceleration, receive‐only coils with 4 channels	

Then, using the visual scoring system based on RAMRIS,^31^ each anatomical region was scored independently by two trained readers who were blinded to clinical data. For tenosynovitis (TSY) and synovitis (SYN), the readers provided a grade on a scale of 0–3, based on the estimated maximum width of peri‐tendinous effusion and synovial effusion/proliferation (for TSY and SYN, respectively) with contrast enhancement,^31^ as follows: grade 0, normal; grade 1, ≤ 2 mm; grade 2, > 2 mm and ≤ 5 mm; grade 3, > 5 mm. The scoring region was bounded proximally by the distal radius/ulna and distally by the hook of the hamate. For bone marrow edema (BME), the readers provided a grade also on a 0–3 scale based on the estimated fraction of affected bone volume: 0, no BME; 1, 1% –33% of bone edematous; 2, 34% –66%; 3, 67% –100%. In the following, the mean scores of the two readers are regarded the ground truth and reference for comparison. Based on the above ground truth scores, the inflammation score of each relevant anatomical structure was obtained and served as the training target. However, since it is extremely rare for most anatomical structures to be affected by inflammation simultaneously, this results in a long‐tailed distribution of the inflammation scores for individual anatomical structures. Consequently, most evaluation metrics are biased and inaccurate, and therefore the evaluation was based on the total inflammation severity ‐ the sum of these scores.

**FIGURE 1:**
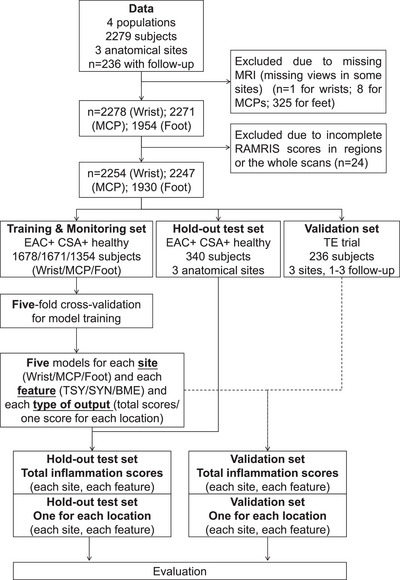
Overview of patient selection within the dataset and the data flow during model training and evaluation.

Twenty‐five subjects (21 EAC patients, three CSA patients and one TE trial patient, respectively) were excluded from this study, due to incomplete visual scores or missing MRIs. Subjects' characteristics, technical information (after selection) and dataflow are shown in Table 1, 2 and Figure 1, respectively.

The whole dataset was then split on a subject level, into a training‐monitoring set (1684 wrist scans/1677 MCP scans/1362 MTP scans from EAC, CSA and healthy controls), a hold‐out test set (340 scans of all anatomical regions from EAC, CSA and healthy controls) and an independent validation set (TE trial); details can be found in 1. Under this split, the training‐monitoring set also included subjects with missing MRI scans on some of the anatomical regions to maximize the amount of information. The hold‐out test set and independent validation set included the patients from whom all anatomical regions were available to fairly compare the performance of models among each region.

To thoroughly evaluate the proposed method and adapt to different uses of the automatic inflammation assessment, the proposed method has two different routes for each input in order to serve different purposes – (1) Route1 serves for the situation that a total inflammation score for each inflammatory sign (TSY/SYN/BME) in the entire anatomical region is needed; (2) Route2 provides extra information on each relevant anatomical structure, it will firstly estimate one score for each inflammatory sign on each anatomical structure (e.g., tenosynovitis severity around the extensor carpi ulnaris tendon) in the anatomical region (e.g., the wrist), and then sum these estimations to calculate the total inflammation score (e.g., tenosynovitis score in the wrist).

To test the robustness of the training process, we applied five‐fold cross‐validation on the training and monitoring sets to obtain five sets of weights (for each anatomical structure, each inflammatory sign) trained with different data in training sets for the same model architecture. Then the models with the different weights were applied to the hold‐out test set and the independent validation set. The robustness of the training process, as the influence of training results on the performance on unseen data, can therefore be checked through the standard deviations in the performance on the evaluation set, using these models with different sets of weights.

### 2.2 Preprocessing

The preprocessing in this ADMIRA system consisted of background removal and slice selection, both were applied to improve the information density of inflammatory signs in the 3D MRI scans. The background removal followed a similar idea in our previous work in rheumatoid arthritis prediction based on MRI scans,^32^ excluding the background which may contain some high‐intensity MRI artefacts or textures. During this background removal, images were (1) thresholded at 10% of the maximum intensity value (optimized manually on a small subgroup of this dataset) to obtain initial foreground masks; (2) then these foreground masks were processed through morphological opening and closing operations^33^, ^34^ to include most of the relevant structures on the borders of the images; (3) subsequently, these foreground masks were applied to the original images to filter out the background; (4) finally, the filtered images were normalized individually and slice‐by‐slice to a range between 0 and 1, with a 95% clipping to avoid over‐normalization caused by the extremely high values from inflamed regions.

The slice selection was applied after the background removal to the filtered images to minimize the influence of background artefact. For each slice in the coronal scans, the standard deviations of the intensities were calculated, and subsequently seven consecutive slices were selected with the highest standard deviations. For the transversal scans, the central seven slices were selected as they typically fell into the scoring areas in manual scoring systems.

### 2.3 Model architecture and training

**FIGURE 2:**
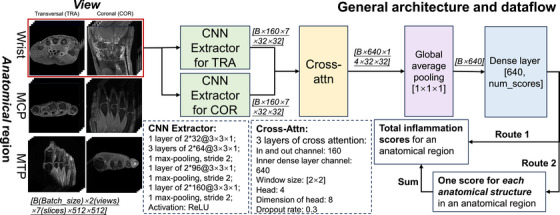
The model architecture and data flow.

To fuse the information from two views (transversal and coronal), as experts intrinsically do during visual scoring, we applied cross‐attention modules.^35^, ^36^ The architecture (see in Figure 2) starts with two separate convolutional neural networks (CNNs) that extract features from the seven slices from the separate transversal and coronal scans. Subsequently, we applied cross‐attention modules to exchange the information extracted from the two views. At the end of the cross‐attention modules, we used adaptive average pooling and dense layers to obtain the inflammation estimations.

3D images with two views (transversal and coronal) of size [2(view), 512, 512, 13 ± 7] voxels were preprocessed to [2, 512, 512, 7] as input during training. The models were trained to output the inflammation scores on each anatomical region by estimating manual scores, and quantifying the inflamed joints.

As introduced above, two routes were defined in ADMIRA system for different clinical uses: (1) for the situation that the total inflammation assessment is needed, Route1 provides a direct estimation for the total inflammation scores (e.g., tenosynovitis for wrists); (2) for the situation that requires an inflammation severity score of each anatomical structure in the anatomical regions (Wrists/MCPs/MTPs), a model is trained to firstly output an estimation for each anatomical structure (e.g., tenosynovitis severity around the extensor carpi ulnaris tendon) and then output the estimation for the entire anatomical region in Route2. Therefore, in total 18 models (3regions×3inflammatorysigns×2typesofoutputs) were trained from scratch, using Kaiming initialization^37^ on the training‐monitoring set, yielding a region‐wise estimation (e.g., total MCP tenosynovitis score) or a structure‐specific estimation (e.g., tenosynovitis score around the extensor carpi ulnaris tendon). The details of the configurations can be found in Table 3.

**TABLE 3:** Configuration of model architecture and training.

Modules & hyperparameters	Configs.
3D Conv Block (1th block)	Conv: depth: 2, kernel size: [3×3×1], number of kernels: 32, stride: 1, group: 2 Activations: ReLU
3D Conv Block (2‐4th block)	Conv: depth: 2, kernel size: [3×3×1], number of kernels: 64, stride: 1 Pool: pooling size: [3×3×1], max pool, stride: 2, group: 2 Activations: ReLU
3D Conv Block (5‐6th block)	Conv: depth: 2, kernel size: [3×3×1], number of kernels: 96 (5th block) and 160 (6th block), stride: 1, group: 2 Pool: pooling size: [3×3×1], max pool, stride: 2 Activations: ReLU
Cross‐attention block (7th block)	In and out channel: 160 Inner dense layer channel: 640 Window size: [2×2] Head: 4 Dimension of head: 8 Dropout rate: 0.3
Adaptive average pooling	Output size: [1×1×1]
Dense layer	Layer 1: [640×2 (views)×1×1×1, number of scores]
Optimizer	AdamW
Learning rate	4e‐5
Weight decay	1e‐2
Optimizer momentum	0.9, 0.999
Training epochs	120
Gradient clip	None

### 2.4 Testing, validation and statistical analysis

Images in the test and independent validation sets were preprocessed through the same pre‐processing methods as during training, and then used as input for the trained models to generate the scores. For each anatomical region and each inflammatory sign, two models with ten different weights (five for each type of outputs) from the training phase was applied to obtain the final scores. The correlation coefficients were calculated based on the total inflammation scores to have a large range of score distribution and minimize the influence of the long‐tailed distribution, as the visual scores of each anatomical structure in anatomical regions are integers between 0 and 3 and the vast majority of them are zero. Furthermore, in pre‐experiments, the mean squared losses between DL outputs and ground truths presented low consistency with the correlation coefficients calculated separately for each anatomical structure during training, leading to low training efficiency and poor performance. Therefore, we chose to evaluate based on based on the sum of all structures in each region, for both types of output that have one score for total inflammation and the type of outputs that have one score for each anatomical structure in a specific anatomical region. Consequently, the score range in the calculation has changed from between 0 and 3 to between 0 and 3x, where x represents the number of defined anatomical structures.

The performance of the proposed method was validated against the performance of human experts, by comparing the agreement between the models' outputs and ground truths with the agreement between readers. This inter‐reader agreement then served as an upper limit for the method's performance, since the ground truth includes the inter‐reader variability. Specifically in this study, we choose the Pearson's correlation coefficients (r) and intraclass correlation coefficients (ICC) between two readers as measures of inter‐reader agreement, since the scores are ordinal data. The definitions of these metrics are shown below. The correlation between two variables $X=[x_{1},x_{2},\cdots ,x_{n}]$ and $Y=[y_{1},y_{2},\cdots ,y_{n}]$ (the scores given by readers or models) is defined as:

(1) $$r=\frac{\sum_{i=1}^{n}\left(x_{i}-\bar{x}\right)\left(y_{i}-\bar{y}\right)}{\sqrt{\sum_{i=1}^{n}{\left(x_{i}-\bar{x}\right)}^{2}}\sqrt{\sum_{i=1}^{n}{\left(y_{i}-\bar{y}\right)}^{2}}}$$

where $\left(x_{i},y_{i}\right)$ are the individual sample points, $\left(\bar{x}=\frac{1}{n}\sum_{i=1}^{n}x_{i},\bar{y}=\frac{1}{n}\sum_{i=1}^{n}y_{i}\right)$ are the sample means of X and Y, n is the number of paired samples (i.e., number of subjects). The ICC used in this study, ICC(2,1),^38^ between two variables $X=[x_{1},x_{2},\cdots ,x_{n}]$ and $Y=[y_{1},y_{2},\cdots ,y_{n}]$ (the scores given by readers or models) can be defined through the following process:

(2) $$\text{ICC}\left(2,1\right)=\frac{2S_{\text{between}}^{2}-S_{\text{error}}^{2}}{2S_{\text{between}}^{2}+S_{\text{error}}^{2}+\frac{2}{n}\left(S_{\text{rater}}^{2}-S_{\text{error}}^{2}\right)}$$

where $S_{\text{between}}^{2}=\frac{1}{n-1}\sum_{i=1}^{n}{\left({\bar{z}}_{i}-\bar{z}\right)}^{2}$ is *Between‐subject variance* that describes variance between subjects, $S_{\text{error}}^{2}=\frac{1}{n-1}\sum_{i=1}^{n}\left[{\left(x_{i}-{\bar{z}}_{i}\right)}^{2}+{\left(y_{i}-{\bar{z}}_{i}\right)}^{2}\right]$ is the *Residual (within‐subject) variance* that describes variance between readers, $S_{\text{rater}}^{2}={\left(\bar{x}-\bar{z}\right)}^{2}+{\left(\bar{y}-\bar{z}\right)}^{2}$ is the *Mean difference between raters* that describes general bias, n is the number of subjects. ICC(2,1) was applied instead of other ICCs because we aimed to evaluate whether $X=\left[x_{1},x_{2},\text{…},x_{n}\right]$ and $Y=\left[y_{1},y_{2},\text{…},y_{n}\right]$ were consistently rated.

Furthermore, to prove the effectiveness of the proposed slice selection and model architecture, we compared the performance with existing methods and the ADMIRA models without the proposed strategies. As the baseline methods in other studies were not originally designed for our image input data and anatomical structures, we used the same pre‐processing (including the slice selection) in these existing methods as in our models, and used an extra dense layer to fuse the information extracted from two input views.

All experiments were executed on an RTX6000 GPU from Nvidia, with PyTorch 1.12 https://pytorch.org/ on Python 3.9 https://www.python.org/ and SciPy 1.7 https://scipy.org/. These were executed ten times with random seeds, and the results of the models with median performance are presented. Details on the configurations and model architectures, configurations and a simple inference application can be found in the online open‐source repository https://github.com/YanliLi27/ADMIRAinfer.

### 2.5 Reliability and explainability

One substantial problem in the deployment of DL models in medical image analysis is their reliability. Although DL models are “black boxes” with large numbers of parameters and difficult to comprehensively ensure their robustness, an intuitive way of facilitating their applications is to improve the explainability. To ensure that DL models in our study were quantifying inflammation based on really inflamed regions instead of artifacts or some other confounders, we applied a revised version^39^ of the class activation mapping (CAM) technique,^40^, ^41^, ^42^ one of the most common explainability techniques for deep learning, to open the black box. The revised method, called rescaled regression activation mapping,^39^ generates heatmaps that highlight the most informative regions, which were expected to be the inflamed regions.

## 3 RESULTS

The ADMIRA system obtained mean ICCs of around 0.9 and 0.8 on the test set and validation set, respectively, for all anatomical regions and inflammatory signs. The same numerical results were obtained in terms of Pearson correlations. For each inflammatory sign and anatomical structure, two outputs are given by the two routes: one total score (synovitis/tenosynovitis/BME) for the whole anatomical region (wrists/MCPs/MTPs) and a series of scores for each anatomical structure (e.g., WRTSYI for tenosynovitis in wrists, MCSYN3 for synovitis in MCPs). Therefore, 18 outputs (three inflammatory signs × three anatomical structures × two routes) are presented in the following sections. The results of the proposed inflammation estimation method is firstly compared with the performance with human experts for indicating the general performance, and then compared with the other DL frameworks and models to show the effectiveness of the whole framework. The ADMIRA system takes a median runtime of less than 0.1 s per input under the training soft‐ and hardware setting, and nearly 1 s per input using normal CPUs.

### 3.1 Performance of route 1 and comparison with human experts

**FIGURE 3:**
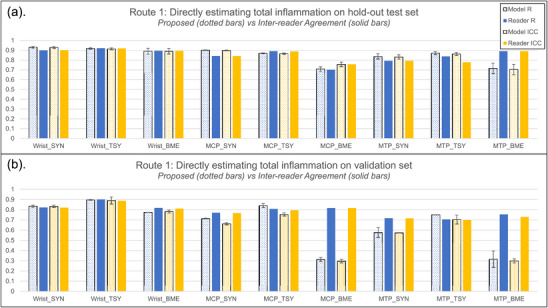
The performance of the ADMIRA system on directly estimating total inflammation scores on (a) hold‐out test set and (b) independent validation set. The dotted bars represent the performance of the proposed method and the solid bars represent the inter‐reader agreement; the blue bars represent the correlation coefficient, R, and the orange bars represent the intra‐class correlation coefficients (ICCs). The error bars on the dotted bars represent the training robustness, derived from the models with different weights during the five‐fold cross‐validation training process on training and monitoring set. The results demonstrate that the trained model based on Route1 performed close to human experts on assessing synovitis and tenosynovitis, yet worse on assessing bone marrow edema. On both sets, the predicted values are statistically correlated (p‐value less than 0.05) with the true values when estimating synovitis and tenosynovitis. The statistical significance on BME vary according to the objects (p‐value from less than 0.0001 on wrist in the hold‐out test set to 0.3 on MTP in the validation set).

Figure 3 presents the general performance of the ADMIRA system on estimating total inflammation scores for the whole anatomical region, on both the hold‐out test set (with a similar inflammation distribution as the training and monitoring sets) and independent validation set (with a different inflammation distribution).

As shown in Figure 3(a), the ADMIRA system received Rs and ICCs around 0.9 on estimating total inflammation scores of synovitis, tenosynovitis and BME in wrists and (teno‐)synovitis in MCPs, while receiving Rs and ICCs of nearly 0.8 for BME in MCPs and tenosynovitis in MTPs. By comparing the inter‐reader agreement on these total inflammation scores according to the manual scoring system, the ADMIRA system achieved a level close to the expert level on the hold‐out test set, especially on estimating tenosynovitis and synovitis.

From Figure 3(b), compared to the performance on the hold‐out test set, the proposed method received Rs and ICCs of around 0.8 for inflammation in wrists, (teno‐)synovitis in MCPs and tenosynovitis in MTPs. However, the ICCs on estimating BME for MCPs and MTPs dropped from 0.7 to 0.3 while the Rs remained around 0.8 and 0.7. These results indicate that the inflammation assessment on tenosynovitis and synovitis is promising, while the assessment on BME based on the ADMIRA system needs further investigation before application.

### 3.2 Performance of route 2 and comparison with human experts

**FIGURE 4:**
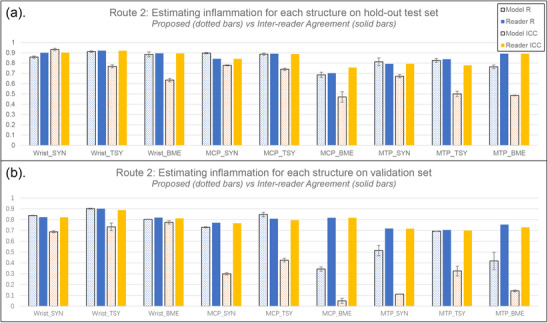
The performance of the ADMIRA system on estimating inflammation scores through summing up the estimated inflammation scores of each anatomical structure on (a) hold‐out test set and (b) independent validation set. The dotted bars represent the performance of the proposed method and the solid bars represent the inter‐reader agreement; the blue bars represent the correlation coefficient, R, and the orange bars represent the intraclass correlation coefficients (ICCs). The error bars on the dotted bars, from the models with different weights during the five‐fold cross‐validation training process on training and monitoring set, represent the training robustness. The results demonstrate that the trained model based on Route2 performed close to human experts on assessing inflammation in the wrist, yet worse on assessing MCP and MTP joints if the distribution of inflammation severity is different. On both sets, the predicted values are statistically correlated (p‐value less than 0.05) with the true values when estimating synovitis and tenosynovitis. The statistical significance on BME vary according to the objects (p‐value from less than 0.001 on wrist in the hold‐out test set to 0.5 on MTP in the validation set).

As mentioned, Route2 firstly generates a score for each anatomical structure in the anatomical region and then sum these scores to obtain the total inflammation scores. Figure 4 presents the general performance of ADIMRA Route2 on both hold‐out test set and independent validation set. The numbers of scores for each inflammation sign and anatomical region are: 10 for wrist tenosynovitis, three for wrist synovitis, 15 for wrist BME, eight for MCP tenosynovitis, four for MCP synovitis, 8 for MCP BME, 10 for MTP tenosynovitis, five for MTP synovitis and 10 for MTP BME.

As shown in Figure 4, the performance of the ADMIRA system on estimating inflammation scores through Route2 – firstly estimate a score for each anatomical structure and then summing the estimates to obtain the total scores – achieved a promising level, with Rs and ICCs around 0.9 on the hold‐out test set and 0.8 on the independent validation set. The ICCs on estimating synovitis and tenosynovitis in the validation set significantly decreased due to a long tail distribution of the inflammation scores. Generally, compared to the performance of Route1 that directly estimate total inflammation scores for the entire anatomical region, Route2 is not as competitive as Route1 in MCPs and MTPs, especially when estimating inflammation scores for MTP and BME. However, Route2 provides the estimation for each anatomical structures in the anatomical regions, enabling more clinical uses that requires the inflammation severity for specific structures.

### 3.3 Output distribution of ADMIRA inflammation assessment

**FIGURE 5:**
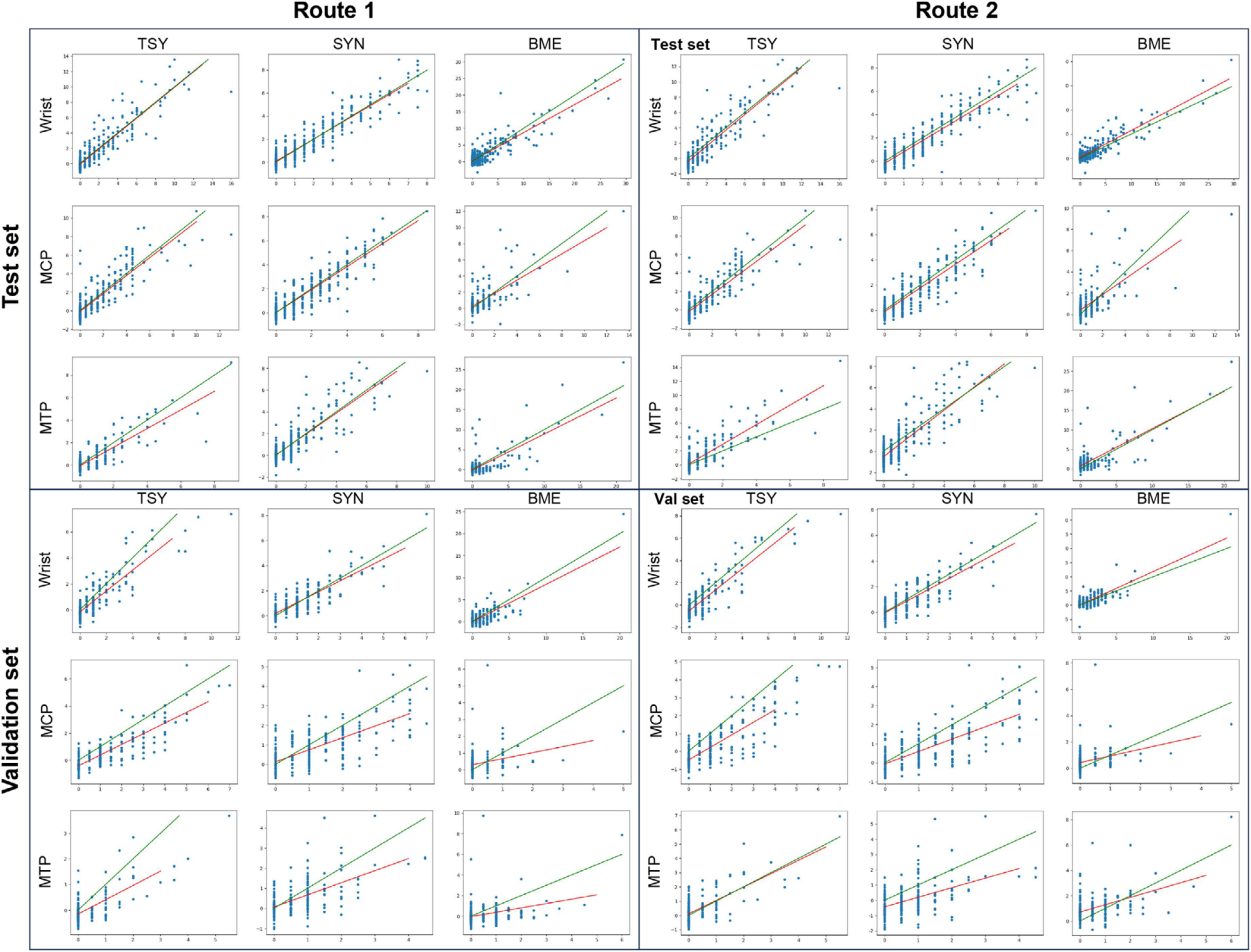
Scatter plots of the ground truths (X‐axis, the mean scores of two readers) and the estimated scores from the ADMIRA system (*y*‐axis) when estimating total inflammation scores through Route1 and when estimating inflammation through Route2. The green diagonal lines represent the ideal relation and the red lines represent the fitted line of the proposed method.

Figure 5 present the scatter plots of the ground truths (*x*‐axis) and the estimated scores from the proposed method (*y*‐axis) of Route1 and Route2, respectively. In these scatter plots, the green diagonal lines represent the ideal correlation, and the red lines represent the fitted line of the proposed method. Generally, from the perspective of inflammatory signs, the proposed method performed the best on estimating tenosynovitis and worst on estimating BME; regarding anatomical regions, the ADMIRA system performed the best on wrists and worst on MTPs; in terms of datasets, ADMIRA performed better on the hold‐out test set than on the independent validation set – probably because the hold‐out test set has an inflammation distribution close to the training and monitoring set. The distribution of errors over the different inflammation severities is relatively uniform, except for some outliers in estimating BME.

### 3.4 Comparison with existing methods

**FIGURE 6:**
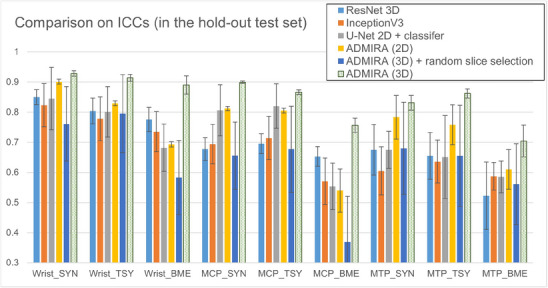
Comparison with baseline methods regarding ICCs and total inflammation estimation (Route1) on hold‐out test set, including ResNet 3D,^30^ InceptionV3,^15^, ^18^ U‐Net encoder with a classifier,^43^ ADMIRA with 2D convolutional blocks, our 3D model with a random slice selection. Except for the 3D model + random slice selection, all models were fed with seven selected slices and the slice dimension served as a channel for the 2D models and as the third dimension for the 3D models. Complete MRI volumes as input were tested and received lower performance compared to slices as input, and was therefore not presented. Our method with 3D models (fed with selected slices) yielded a significantly greater similarity compared to other methods, as supported by a p‐value less than 0.05.

Figure 6 presents the comparison of the proposed method and some existing methods. As these baseline methods were not designed for the input and the anatomical objects, we used the same pre‐processing (including the slice selection) as we applied to our models and used an extra dense layer to fuse the information extracted from two input views. In addition, Figure 6 presents the results using the same architecture with 2D Convolutional and 2.5D inputs and using random slice selection to show the effectiveness of the proposed preprocessing and 3D inputs. The results between our model with and without the slice selection demonstrate the effectiveness of the proposed preprocessing. Meanwhile, the comparison with other model architectures indicates the effectiveness of the Transformer‐based information fusion. The comparison between the 2D and 3D version of ADMIRA suggest 3D inputs with 3D Conv could preserve more information than 2.5D inputs and 2D Conv.

### 3.5 Explainability through CAM

**FIGURE 7:**
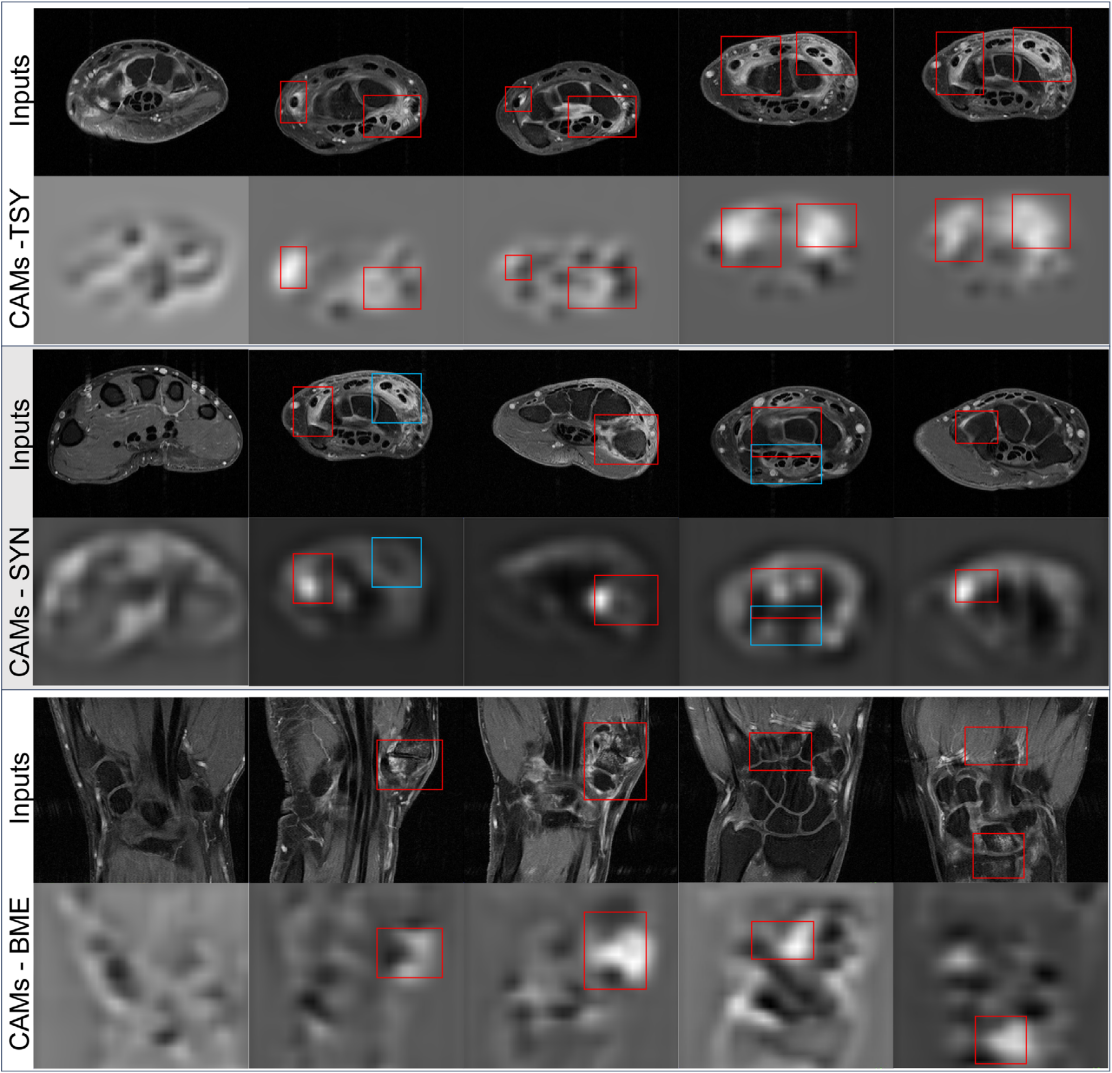
Examples of slices from the CAMs of 15 patients generated from the DL models for estimating total inflammation directly (Route1). Red boxes in the examples highlight the inflammation regions in the MRI scans that are the target regions for inflammation assessment; blue boxes highlight examples of inflamed regions that belong to another category (e.g., tenosynovitis regions when the CAMs were generated from the DL models for synovitis estimation). CAMs are displayed as separate images instead of the commonly used overlays on the original images, in order to allow for an accurate interpretation by clearly distinguishing class activations from pixel intensities.

Figure 7 presents some examples of slices in the CAMs generated from the DL models in ADMIRA trained for the estimation of total inflammation score directly (Route1). The visual checks of the CAMs suggest that the DL models of ADMIRA followed a principle similar to visual scoring, and to some extent proved the reliability of the proposed method. Moreover, the CAMs also suggest some potential correlation between different inflamed regions, as tenosynovitis regions received higher signal intensities compared to the background, and the intensities of the background should represent no contribution to the evaluation of inflammation.

## 4 DISCUSSION

To our knowledge, this ADMIRA is the first study to develop an end‐to‐end automatic tool for joint inflammation scoring of rheumatoid arthritis with such a performance close to human experts. The 3D version of ADMIRA system successfully measured joint inflammation in three anatomical regions, achieving high ICCs with the ground truth from visual scoring on fat saturated, contrast‐enhanced T1‐weighted MRI scans. The automatic estimations of tenosynovitis and synovitis achieved an accuracy close to that of expert assessments, while the challenges in estimating BME require further investigation and validation.

### 4.1 Data distribution

**FIGURE 8:**
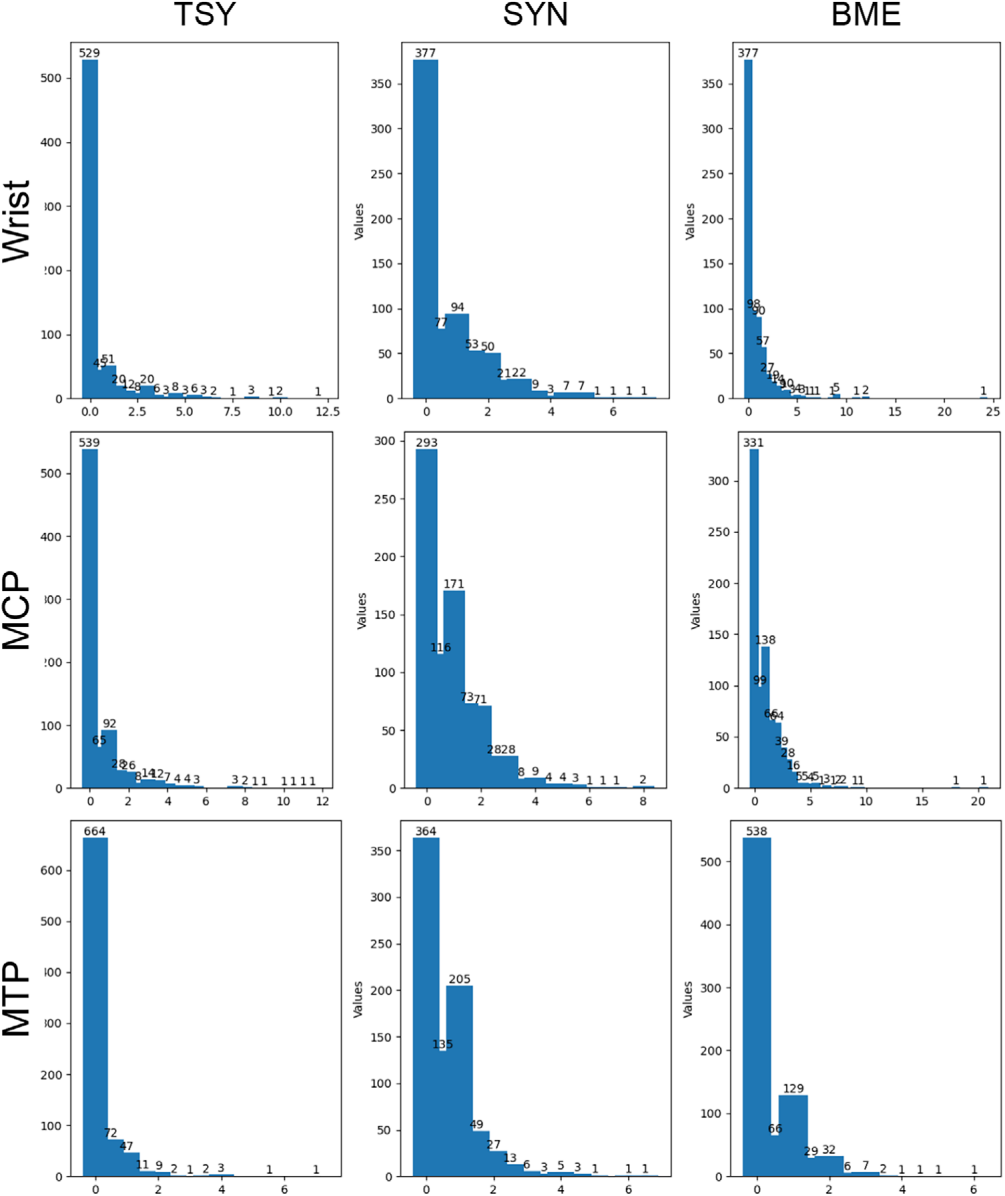
The inflammation score distribution of each inflammatory sign in the group of validation set.

The relatively poor performance in estimating BME may stem from two main factors: (1) the image features of BME in MRIs (such as intensity and boundary) are not as clear as synovitis or tenosynovitis, therefore making it difficult for the method to learn consistent patterns of BME; and (2) the estimation of BME faces a considerable imbalance in the distribution of inflammation severity across the whole dataset. Most MRI scans in all the populations studied received a score between 0 and 1 in each anatomical structure and between 0 and 3 in total score, based on RAMRIS. This imbalanced, long‐tailed distribution (see Figure 8) affected the training of DL models and increased the difficulty of obtaining high ICCs and Rs. Some compensations such as data augmentation and over‐sampling were conducted to mitigate this distribution problem. However, inflammation in each anatomical structure is not evenly distributed (some anatomical structures are more susceptible to inflammation) and sometimes correlated with each other, the compensations were not enough to solve the problem. A potential solution to this data imbalance is advanced data augmentation^11^ or synthesis^44^ that controllably over‐sample certain rare cases.

Similar factors also affected the performance of the ADMIRA system on the independent validation set, where the distribution of inflammation severity significantly differed from that of the training, monitoring and hold‐out test sets. This discrepancy across the independent validation set derived from the inclusion criteria of the TE trial – TE trial only consists of patients with more subclinical joint inflammation than the healthy controls and the patients included in CSA, and more importantly, the patients with most severe joint inflammation were not included. These different inclusion criteria lead to more patients with only mild joint inflammation and less patients with severe joint inflammation in TE trials, compared to the other study populations, in which EAC patients with severe joint inflammation were included and mitigated the influence of long‐tailed distribution. Consequently, the visual scores for all kinds of joint inflammation in the independent validation set present a longer‐tailed distribution that undermines the performance.

The long tail distribution also explains why ADMIRA performed worse in estimating the scores for each anatomical structure than estimating the total scores directly. The visual scores for each anatomical structure across all scans and all anatomical regions (wrists, MCPs and MTPs) presented severely long‐tailed distributions: for each anatomical structure, the score was 0 in the vast majority of scans. The DL models trained to estimate the visual scores for each anatomical structure were therefore facing a similar data imbalance and received moderate performance compared to the models estimating the total scores directly.

### 4.2 Limitations

Besides the consequences of the imbalance in data distribution, the limitations of ADMIRA are of great importance to discuss. The first limitation is that the method is designed for the described extremity 1.5T MRI protocol with contrast agent administration. While the ideas can be transferred to other MRI sequences or field strengths, some specific adaptations and refinements are required for application to other protocols, and the effectiveness of these ideas cannot be ensured on those datasets without challenges similar to our tasks.

The second limitation is that the performance of DL models may be overestimated due to the potential correlation among inflamed regions, leading to an inflammation estimation on one anatomical structure that originates from a prediction based on another inflamed region instead of the target region.

The third limitation comes from the fundamental basis of the model training, in which visual scores were considered as the ground truths. On the one hand, RAMRIS scores are the best reference we could access in assessing joint inflammation based on MRI scans; on the other hand, training on these visual scores as golden standards has inherent drawbacks: (1) RAMRIS has a semi‐quantitative scale, ignoring subtle differences in scores of less than 1 unit, that could have negative impact on training a model with continuous outputs; (2) Some inflamed tissues are not included in the RAMRIS as it is designed for rheumatoid arthritis, limiting the application range of the proposed method; and (3) The proposed method cannot surpass the performance of RAMRIS, and the quality of ground truths (e.g., inter‐reader agreement, see in the appendix) used for training could convey implicit biases and influence the reliability of the proposed method.

The fourth limitation derives from the MRI acquisition protocol. ADMIRA was trained based on fat saturated, contrast‐enhanced T1‐weighted MRI scans. Consequently, its ability on MRI scans without contrast agent requires further investigation. As contrast‐enhanced MRI scans are not always available,^44^ the ability of detecting and assessing inflammation in MRIs without contrast agent is therefore of more significant implications. Therefore, we are aiming at developing a model based on MR images without contrast enhancement using the current model as a pretrained model in the following projects.

Furthermore, the issue on explainability caused by choosing end‐to‐end training is a major disadvantage of the proposed DL method, compared to the previous segmentation‐quantification approach^11^, ^45^ or landmark‐quantification approach.^29^ This disadvantage also affects the applications of other methods that are aiming at end‐to‐end score estimation, as mentioned in introduction. To validate the DL models, we applied the improved regression activation mapping algorithm to generate heatmaps that highlight important regions in MRI scans during the estimation of inflammation severity based on DL models. To some extent, the explainability of the DL models could be verified by comparing the anatomical structure of highlighted regions in the heatmaps and the inflamed regions, proving DL models were estimating inflammation correctly based on the truly inflamed regions in the MRI scans. However, evaluating the reliability by an observer study faces challenges including the experiment design and comprehensive understanding of both in‐depth clinical and technical details. For example, as deep learning models can learn from the correlation in inflammation between two regions, the CAMs may also highlight multiple regions out of the ROIs, leading to difficulties in a comprehensive observer study. Considering the importance of the explainability, we are currently working on a comprehensive clinical study in cooperation with clinicians on the reliability, generalizability, efficiency and other perspectives of the proposed method.

### 4.3 The two routes for inflammation estimation

The two routes in this study to obtain the inflammation estimation are designed for different purposes. For clinical uses that requires inflammation assessment for some specific structures in the anatomical regions, Route2 provides a structure‐wise estimation that can be flexibly selected, summed and then applied to different aims. For the situation that only the total inflammation severity is needed, Route1 enables a more accurate estimation, allowing the model to infer and “predict” total inflammation based on any inflammatory signs in the images without precisely estimating inflammation of each structure.

### 4.4 Summary

Despite the above limitations and the need for a comprehensive observer study for explainability, the end‐to‐end DL models brought not only expert‐level accuracy on inflammation estimation, but also a fast calculation in less than 0.1 s (GPU) and 1 s (CPU) for each input and independency on accurate manual annotations or landmarks. These advantages allow the proposed method to be performed on other similar clinical problems and datasets, contributing to reducing the labor and time costs of inflammation assessment.

## 5 CONCLUSION

In this study, we proposed a fully automatic system for scoring inflammation from extremity MRI scans called ADMIRA, quantifying the inflammatory signs in (teno‐)synovia and bones of the wrists, MCPs and MTPs. The deep learning model learned the meaning of inflammatory areas on fat saturated, contrast‐enhanced T1‐weighted extremity MRI scans and provided accurate and fast inflammation scoring for wrists, MCPs and MTPs, especially on synovitis and tenosynovitis, based on principles similar to human assessments. We expect that this automatic method could help to reduce labor costs and improve the efficiency of diagnosis in the future.

## CONFLICT OF INTEREST STATEMENT

The authors have no relevant conflicts of interest to disclose.
